# Acute Cd Toxicity, Metal Accumulation, and Ion Loss in Southern Catfish (*Silurus meridionalis* Chen)

**DOI:** 10.3390/toxics9090202

**Published:** 2021-08-29

**Authors:** Wenming Liu, Hanxun Qiu, Yulian Yan, Xiaojun Xie

**Affiliations:** Key Laboratory of Freshwater Fish Reproduction and Development, Ministry of Education, School of Life Sciences, Southwest University, Chongqing 400715, China; qiuhx302315@email.swu.edu.cn (H.Q.); yanyul@swu.edu.cn (Y.Y.)

**Keywords:** cadmium, acute toxicity, accumulation, bioconcentration factor, calcium, sodium, *Silurus meridionalis*

## Abstract

The amounts of cadmium in multiple organs and the amounts of Na^+^ and Ca^2+^ in the carcass were measured in dead and surviving southern catfish exposed to different concentrations of Cd. The 96 h median lethal concentration was 6.85 mg/L. The Cd content and Cd accumulation rate were positively correlated with Cd exposure concentrations, and there were significant differences between dead and surviving individuals, indicating that both Cd content in tissues and Cd accumulation rates were correlated with mortality. Cd levels in the liver of dead fish were saturated. A lethal threshold for Cd concentration in the whole fish was obtained. Bioconcentration factors for Cd did not decrease with increasing exposure. Acute exposure to waterborne Cd caused a significant decrease in the ion content of the fish carcass. There was a significant difference between the Na^+^ content of the carcass of dead fish (34.54 μmol/g wet weight) and surviving fish (59.34 μmol/g wet weight), which was not the case with the Ca^2+^ content, indicating that the lethal toxicity of Cd was probably related to the decrease in Na^+^ content. Collectively, these results suggest that whole-fish Cd concentration and carcass Na^+^ content can be useful indicators of fish acutely exposed to Cd.

## 1. Introduction

Anthropogenically derived impacts have unequivocally contributed to environmental poisoning by metals in aquatic ecosystems during the last few decades [1]. Among these heavy metals, cadmium (Cd) is of particular concern because of its widespread distribution in the aquatic environment [2,3], and because it is highly toxic to both fish and invertebrate marine organisms [4,5,6,7]. Heavy metals have a bioaccumulative nature. Bioaccumulation means the accumulation over time of a substance, especially a contaminant (such as a heavy metal), in a living organism [8]. Cd tends to accumulate in fish and invertebrate marine organisms [7]; as fish and invertebrate marine organisms represent the main contaminated foods that are consumed by humans, Cd is indirectly becoming toxic to human beings [7]. In China, the Cd concentration in normal fresh water ranges from 10 ng/L to 8 μg/L [9]; Cd and Cd concentrations in polluted water range from 0.8 to 12.05 mg/L [9,10]. Cd pollution incidents are frequently found in aquatic systems in China such as that in the Guangdong North River in Shaoguan city in 2005 and the Longjiang Cd pollution event in Hechi, Guangxi in 2012 [11]. Cd emissions have occurred occasionally, so cadmium exposure is a real danger to aquatic organisms, including fish [12,13]. Cd pollution will have a negative impact on the fitness and survival of aquatic organisms, as indicated by a decrease in biodiversity of polluted areas [10]. Cd accumulation in fish can cause a variety of toxicities, including ion regulation disorders, oxidative damage, endocrine disorders, genetic toxicity, histopathological changes, and can even result in the death of fish [14,15,16,17,18,19].

The acute or chronic intoxication of organisms and a variety of adverse effects from Cd have emerged as a global environmental threat to aquatic organisms and aquatic ecosystems. Toxicity to fish resulting from acute exposure to Cd in water has been extensively studied [15,20,21]. These studies have mostly reported 96 h median lethal concentrations, indicating the sensitivity of different fish species to Cd exposure in water. However, even under controlled conditions, the LC_50_ (median lethal concentration) can be affected by external physical, chemical, and biological factors in the aquatic environment [22,23]. A query in the EPA ECOTOX database [24] for 96h LC_50_ (a 96 h median lethal concentration) values of Cd toxicity for zebrafish (*Danio rerio*) in recent literature found a wide range of concentrations: 96 h LC_50_ for Cd was between 3822 and 22,482 μg/L [14,25,26]. The concentration of accumulated heavy metals in target sites of fish organs and tissues is closer to the actual concentration of heavy metal toxicity and is not easily affected by external factors. Therefore, the tissue residue approach (TRA) was proposed [27], which uses tissue concentration as a more reliable toxicity indicator, and its application in environmental toxicology studies is of great value for the protection of species and the ecological environment [28,29,30,31]. Current studies on the toxicity of Cd according to TRA have mainly focused on aquatic oligochaetes [22,32,33], and there is a lack of studies in fish [34]. Although studies have compared the difference between whole-fish Cd content in dead and surviving fish under acute waterborne Cd exposure [35,36], they did not consider looking at the difference in whole fish Cd content under different Cd exposure concentrations, nor studying different tissues to determine if there were differences in Cd content, nor elucidating the quantitative relationship between Cd exposure concentrations and tissue Cd content. Therefore, it is necessary to study the relationship between Cd content and toxicity in different organs and tissues of fish under acute Cd exposure conditions in the water.

Following Cd exposure, the main mechanism of fish toxicity is the disturbance of Na^+^ and Ca^2+^ ion homeostasis [14,36,37,38,39,40,41,42,43]. Whole body Na^+^ levels in zebrafish (*Danio rerio*) and rainbow trout (*Oncorhynchus mykiss*) are commonly used as indicators for acute and subacute Cd^2+^ toxicity in fish [14,15,36]. Most studies on the effect of Cd^2+^ on fish Ca^2+^ levels have focused on blood [37,44], and the toxic effects of Cd^2+^ have only been occasionally studied using the whole body Ca^2+^ level in fish [4,45]. It is not clear whether the principles of Cd toxicity established by these model organisms also apply to other non-model fish [46].

**Figure A1:**
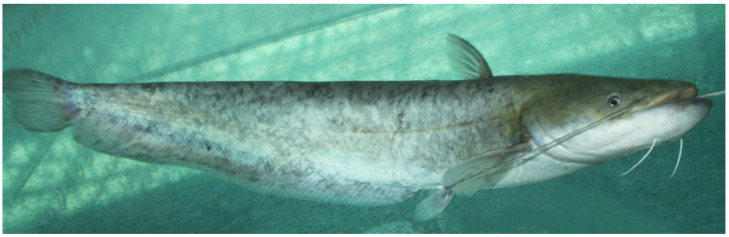
A picture of the southern catfish (*Silurus meridionalis* Chen).

The southern catfish (*Silurus meridionalis* Chen) is a carnivorous fish with a rapid growth rate and high economic value, and it is widely distributed in the Yangtze River and southern regions of China [47] (Figure A1). Southern catfish is commonly consumed as a potentially valuable protein source [48]. Due to the influence of water pollution and other factors, the southern catfish has lost its dominant position in some sections of the Yangtze River [49]. Studies have shown that the southern catfish readily takes up Cd from the aquatic environment, leading to high Cd content in the body although information regarding Cd toxicity in this species is limited. Carnivorous fish have a strong ability to biomagnify Cd in the food chain [50]. The chronic exposure of southern catfish to Cd damages liver mitochondrial structure and function [51], causes oxidative damage to the gills, liver, and kidney tissues [52], and also affects growth and development [53]. However, gaps remain in the knowledge regarding acute exposure and tissue-specific Cd accumulation. In order to protect this economically important food fish, it is necessary to determine the tolerance limit of southern catfish to Cd as a persistent heavy metal pollutant. Acute toxicity testing plays an important role in the sustainable management and conservation of the natural habitats of fish [50]. In addition, fish can take up Cd from both waterborne and dietary sources; foodborne exposure generally leads to higher metal concentrations in the intestines compared with the gills, whereas for waterborne exposure, metal concentrations in the kidneys and the gills are the higher than in the other tissues [54]. Therefore, this study aimed to consider the following acute waterborne Cd exposure conditions for southern catfish: (1) the quantitative relationships between Cd accumulation concentration in different organs and tissues and Cd exposure concentration, (2) the different accumulated Cd concentrations between living and dead fish, and (3) the relationships between Na^+^ and Ca^2+^ content and acute Cd-induced death.

## 2. Materials and Methods

### 2.1. Experimental Water and Reagents

Deionized water (5 μs/cm) was obtained using a water purifier (LD 3000G A2, Chongqing Lidi Experiment Instrument Co., Ltd., Chongqing, China). MgSO_4_∙7H_2_O (AR), KCl (AR), CaCl_2_∙2H_2_O (AR), NaHCO_3_ (AR), and CdCl_2_ (99.995% purity on trace metal basis) were purchased from Solarbio Life Sciences. Nitric acid (HNO_3_, certified reagent grade) was purchased from Chongqing Chuandong Chemical Industry Group Co. LTD (Chongqing, China). Perchloric acid (HClO_4_, certified reagent grade) was purchased from Chengdu Kelong Chemical Co. LTD (Chengdu, China). All glassware and polyethylene vials were kept in 10% HNO_3_ solution overnight and rinsed three times with ultrapure water prior to use.

### 2.2. Experimental Fish and Acclimation

All animal procedures were conducted in accordance with the Institutional Animal Care and Use Committee (IACUC) guidelines of Southwest University (IACUC-20180303-01) and the environment and housing facilities for laboratory animals of China (GB/T 14925–2001). Juvenile southern catfish were acquired from the Institute of Hydrobiology and Water Environments, Southwest University, Chongqing, China. The fish weighed nearly 40 g and were acclimated in a polyethylene recirculating rearing system for 2 weeks, under a photoperiod with 12 h of light and 12 h of dark (12 L:12 D), before the start of the experiment. During the acclimation period, the fish were fed to satiation with fresh pieces of grass carp (*Ctenopharyngodon idella*) once daily at 18:00. About 50% of the water in the recirculating rearing system was renewed daily. The water quality parameters were as follows: temperature 27.5 ± 0.1 °C, pH 7.10 ± 0.2, dissolved oxygen above 7.5 mg/L, ammonia concentration under 0.03 mg/L, and CaCO_3_ hardness of 25.2 ± 0.2 mg/L (OECD 203 annex 2, 1992: MgSO_4_∙7H_2_O 0.0123 g/L, KCl 0.0006 g/L, CaCl_2_∙2H_2_O 0.0294 g/L, and NaHCO_3_ 0.0065 g/L). The study was conducted in accordance with the ethical requirements and recommendations for animal care of the Fisheries Science Institution of Southwest University, China.

### 2.3. Acute Cd Exposure

The experiment was conducted based on guidelines provided by Vergauwen (2013) [25] and Yılmaz (2004) [55]. After two weeks of acclimation, two hundred healthy and similar-sized fish (50.0 ± 0.2 g) were randomly distributed to thirteen 400 L polyethylene recirculating rearing systems. Each system consisted of 20 breeding units (length × width × height = 39 cm × 27 cm × 25 cm). Ten or twenty fish were kept within each recirculating rearing system. However, in order to avoid cannibalism, each fish was kept in a separate breeding unit. The nominal total Cd concentrations were 0 (control), 3, 4, 5, 6, 7, 8, 9, 10, 11, 12, 13, and 14 mg/L for each exposure system. Cd was added as CdCl_2_ (Beijing Solarbio Science and Technology Co., Ltd., Beijing, China). It was dissolved in distilled water to obtain stock solutions of 20 mg/mL Cd^2+^. Desired Cd^2+^ levels were achieved by adding appropriate volumes of the stock solution to the aquarium water. During the test period, the water was renewed (50%) each morning, and the corresponding stock solution of Cd^2+^ was added at the time of the water change. Total Cd concentrations were measured with flame atomic absorption spectrometry (TAS 990, Beijing Purkinje General Instrument Corporation, Beijing, China) and adjusted before the start of the experiment and every 12 h during the experiment to ensure a maximum 10% deviation from the nominal concentrations. The actual measured dissolved Cd^2+^ concentrations (after 0.45 μm filtration) were 0, 2.96 ± 0.13, 3.97 ± 0.18, 5.01 ± 0.11, 5.98 ± 0.23, 7.02 ± 0.05, 8.02 ± 0.06, 8.97 ± 0.06, 10.01 ± 0.06, 11.03 ± 0.10, 11.98 ± 0.06, 13.01 ± 0.02, and 14.02 ± 0.05 mg/L (n = 8), respectively. The fish were starved two days prior to the experiment and did not feed throughout the acute toxicity trials. During the test period, the temperature, pH, dissolved oxygen concentration, ammonia-nitrogen concentration, water hardness, and photoperiod were identical to those used during acclimation. In order to minimize the effects of continued immersion in tanks on the amounts of Cd^2+^, Ca^2+^, and Na^+^ in dead fish, the fish were taken within 1 h after death. Mortality and time to death were recorded every 1 h over 96 h, and dead fish were immediately removed, rinsed in Cd-free reconstituted water, then sampled. After 96 h, fish that survived the toxicity test were rinsed in Cd-free reconstituted water, then sampled. The gills, liver, kidneys, and heart were removed, weighed, and saved, as was the remaining carcass. The gills were vigorously rinsed in deionized water and blotted dry immediately after removal. All samples were stored at −80 °C until their use for the determination of the tissue Cd concentration and ion (Ca, Na) analysis.

### 2.4. Tissue Cd and Ion (Na^+^ and Ca^2+^) Analysis

After weighing, the tissue samples were digested with HNO_3_ and HClO_4_. The carcasses were dried at 70 °C to a constant weight and then ground to a fine powder with a mortar and pestle. A total of 300 mg of the powder was digested in HNO_3_ and HClO_4_. The digestion process was initiated at room temperature overnight, followed by incubation in a hot block (EH35A Plus, LabTech, Beijing, China) at 120 °C. The resulting solution was evaporated, and the residue was redissolved in 0.5% HNO_3_.

The Cd^2+^ and Ca^2+^ content of all samples was measured by flame atomic absorption spectrometry (FAAS) (TAS-990, Beijing Purkinje General Instrument Corporation, Beijing, China), and the Na^+^ content of the carcasses were measured by flame photometry (AP1500, Aopu Analytical Instruments, Shanghai, China). The quality was assessed for each batch of digested samples, and each batch included blank samples and trace element-spiked samples. The calculated recoveries of the reference material were 98.70%, 96.51%, and 100.04% for Na, Ca, and Cd, respectively.

### 2.5. Calculations and Statistical Analysis

The Cd content was expressed as μg/g wet weight and calculated using measured concentrations and tissue weights, with whole body values representing the sum of measured tissue and carcass contents. Based on data for tissue Cd concentrations, we estimated the average accumulation rates of tissue Cd using the following equation [36]:

Accumulation rates of tissue Cd = (Tissue Cd)/t

where (Tissue Cd) = Cd concentration in the tissue of the southern catfish (μg Cd/g wet weight) collected at the time of death (hour) and the end of the experiment after 96 h; and t is the fish collection time (hour).

The bioconcentration factor (BCF) for tissues were calculated considering the concentrations of Cd determined in the fish tissue (C*_f_*, μg/g wet weight) and in the water in which the fish was raised (C*_w_*, μg/mL), using the equation [56]:

BCF = C*_f_*/C_w_

Data are presented as means ± SEM. For the exposure concentration–response curves, the percentage of mortality after 96 h was presented as a function of the Cd exposure concentration. Normality of data was verified using the Shapiro–Wilk test, and the homogeneity of variances was tested using Levene’s test. Na^+^ and Ca^2+^ content was compared to the corresponding control value using Student’s *t*-test. Analysis of covariance (ANCOVA: with Cd concentration in fish tissues as a dependent variable, Cd exposure concentration as a covariate, and outcome (dead or alive) as a response variable) was used to test for differences in overall Cd accumulation between dead and surviving fish. The analysis of covariance was also used to compare the rates of Cd accumulation. All statistical tests and linear regression estimations, and estimates of 95% CI, were run using GraphPad PRISM 8.0 (GraphPad Software Inc., La Jolla, CA, USA) or IBM SPSS 22.0 (© SPSS Inc., Chicago, IL, USA) at *p* ˂ 0.05. The logistic regression and analysis of covariance were used by IBM SPSS 22.0 (© SPSS Inc., Chicago, IL, USA). Linear regression estimations and graphs were drawn with GraphPad PRISM 8.0 (GraphPad Software Inc., La Jolla, CA, USA). 

## 3. Results

### 3.1. Mortality and 96 h LC_50_

**Figure 1:**
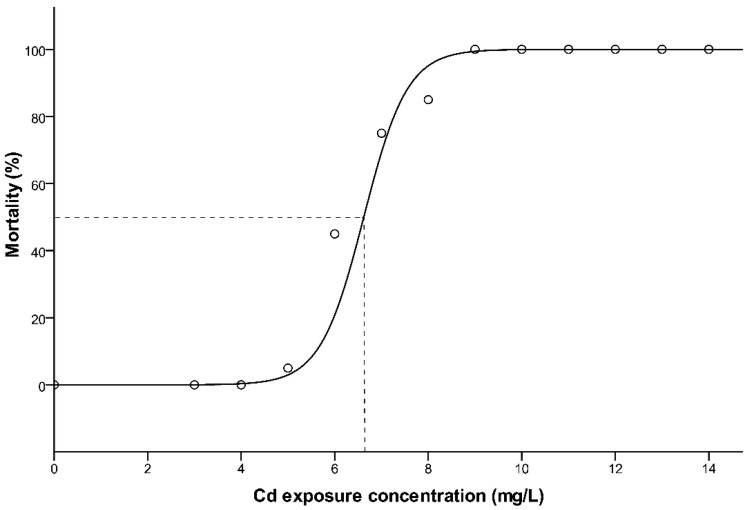
The logistic fit of the model to the dataset of Cd exposure for 96 h. LC_50_ with dotted line in the graph.

The mortality of southern catfish for each Cd exposure are presented in Figure 1. A clear, dose-dependent mortality was induced by Cd exposure test, where higher concentrations caused 100% mortality for all the fish, whereas the tanks spiked with lower doses of Cd were characterized by intermediate to zero mortality. No mortality occurred in the control (0 mg Cd/L). Mortality is plotted as a function of the nominal concentration of Cd (mg/L). The relationship between mortality and Cd exposure concentrations can be fitted by a logistic regression equation (Figure 1), and the regression model equation obtained was *y* = 1/(0.01 + 75,918.77 e^−2.313*x*^), (r^2^ = 0.850, *p* < 0.05) (*y* = mortality, *x* = Cd exposure concentrations, mg/L). Based on this equation, the 96 h LC_50_ value was 6.85 mg/L.

### 3.2. Cd Accumulation and Accumulation Rate Analysis of Tissues

**Figure 2:**
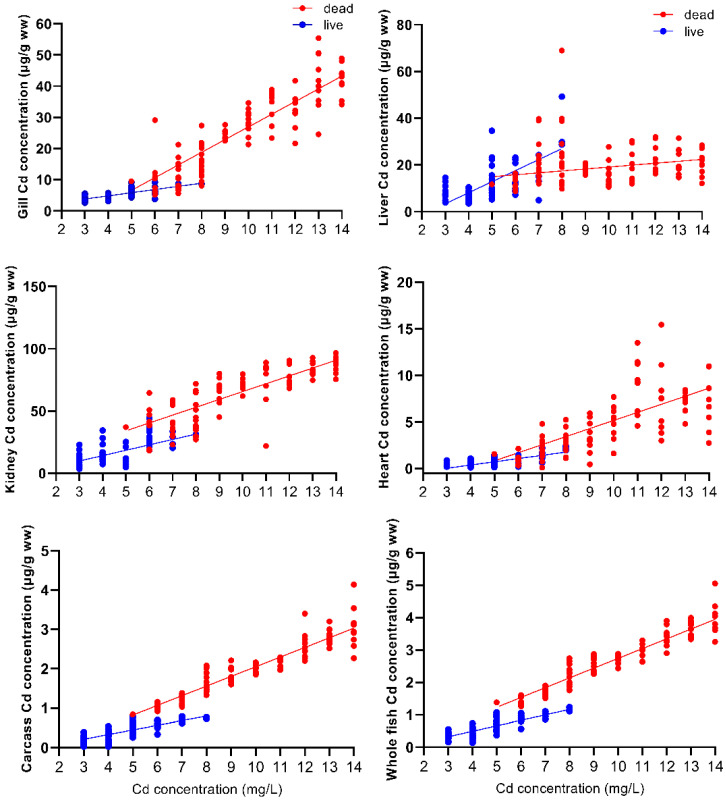
Relationship between the exposure and Cd accumulation concentrations in the gills, liver, kidneys, heart, carcass, and whole fish of *Silurus meridionalis*. Red dots represent fish that died within 96 h, and blue dots represent fish that survived after the 96 h exposure. Regression analyses for the six tissues examined from the 102 fish that died during the 96 h exposure are indicated by the red line. Regression analyses for the six tissues examined from the 78 fish that survived the 96 h exposure are indicated by the blue line.

The relationship between Cd concentrations in different tissues and Cd exposure concentration is shown in Figure 2. There was a positive linear correlation between the Cd concentrations in the gills, kidneys, heart, and carcass of dead fish and the Cd exposure concentration of the whole fish (*p* < 0.05). However, there was no linear correlation between liver Cd accumulation and Cd exposure concentration (*p* > 0.05). The Cd concentrations in all tissues and in surviving whole fish were positively and linearly correlated with Cd exposure concentration (*p* < 0.05). Regression analyses for the six tissues examined from the 102 fish that died during the 96 h exposure are *y* = 4.07*x* − 13.7, r^2^ = 0.96, *p* < 0.0001 (gill); *y* = 0.823*x* + 10.9, r^2^ = 0.38, *p* > 0.05 (liver); *y* = 6.29*x* + 2.72, r^2^ = 0.94, *p* < 0.0001 (kidney); *y* = 0.867*x* − 3.50, r^2^ = 0.82, *p* < 0.001 (heart); *y* = 0.245*x* − 0.402, r^2^ = 0.99, *p* < 0.0001 (carcass); *y* = 0.302*x* − 0.276, r^2^ = 0.99, *p* < 0.0001 (whole fish). Regression analyses for the six tissues examined from the 78 fish that survived the 96 h exposure are *y* = 1.02*x* + 0.764, r^2^ = 0.95, *p* < 0.05 (gill); *y* = 4.73*x* − 10.8, r^2^ = 0.69, *p* < 0.05 (liver); *y* = 4.33*x* − 3.10, r^2^ = 0.95, *p* < 0.05 (kidney); *y* = 0.347*x* − 1.01, r^2^ = 0.74, *p* < 0.05 (heart); *y* = 0.117*x* − 0.138, r^2^ = 0.91, *p* < 0.001 (carcass); *y* = 0.168*x* − 0.178, r^2^ = 0.95, *p* < 0.001 (whole fish), where *x* is the Cd exposure concentration and *y* is the Cd concentration for the respective tissues.

The slope of the accumulation of Cd concentrations in the gills, kidneys, heart, carcass, and whole body of dead fish with Cd exposure concentrations was significantly higher than that for the surviving fish (gill *F* = 68.336, *p* < 0.001; kidney *F* = 6.891, *p* < 0.05; heart *F* = 20.384, *p* < 0.001; carcass *F* = 35.260, *p* < 0.001; whole fish *F* = 44.615, *p* < 0.001). The effect of Cd exposure concentration on the Cd concentration in different tissues and the whole body of dead and surviving fish was also very significant (gill *F* = 179.149, *p* < 0.001; liver *F* = 32.485, *p* < 0.001; kidney *F* = 123.614, *p* < 0.05; heart *F* = 53.761, *p* < 0.001; carcass *F* = 371.862, *p* < 0.001; whole fish *F* = 545.561, *p* < 0.001). There was a clear threshold between the Cd concentration in the dead and surviving fish; in the surviving fish, the accumulation of Cd never exceeded 1.16 μg/g relative to the wet weight of whole fish, but in fish that died of acute Cd poisoning, the accumulation of Cd was a minimum of 1.38 μg/g wet weight of the whole fish (Figure 2).

**Figure A2:**
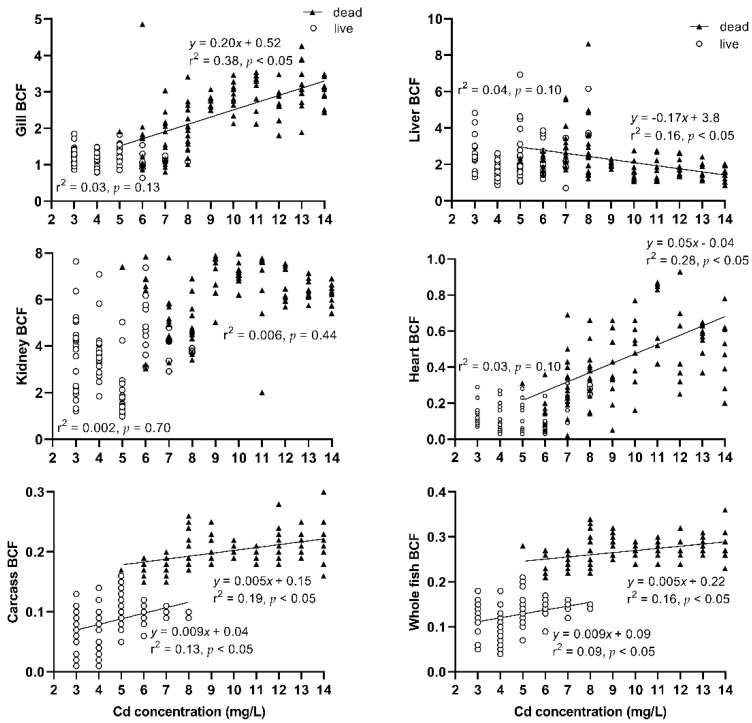
Relationship between exposure concentration and the BCF of gills, liver, kidneys, heart, carcass, and whole fish of *Silurus meridionalis*. Triangles represent fish that died within 96 h, and circles represent fish that survived the 96 h exposure.

Cd bioconcentration factors (BCF) in gill, liver, and kidney tissue were above 1, but in the heart, carcass, and the whole fish, BCF were below 1 (Figure A2). BCF in gill, heart, carcass, and the whole body of dead fish trended towards a positive relationship with exposure (*p* < 0.05), but liver tissue BCF trended towards an inverse relationship with exposure (*p* < 0.05). There were no linear correlations between the BCF in gill, liver, kidney, and heart tissues of surviving fish and Cd exposure concentration (*p* > 0.05). However, BCF in the carcass and whole body of surviving and dead fish trended towards a positive relationship with exposure (*p* < 0.05).

**Figure 3:**
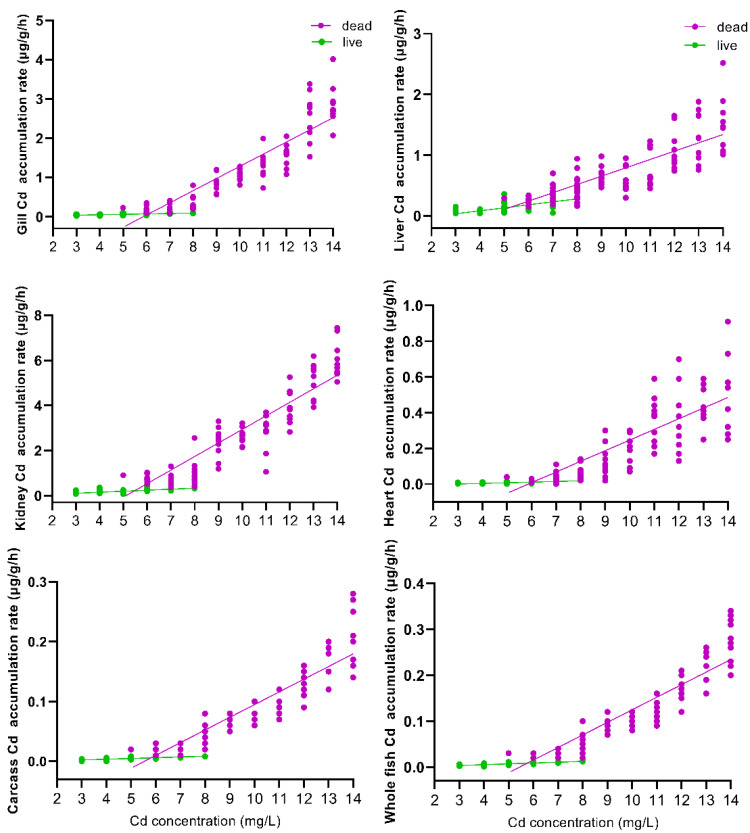
Relationship between exposure concentration and the Cd accumulation rate in the gills, liver, kidneys, heart, carcass, and whole fish of *Silurus meridionalis*. Purple dots represent fish that died within 96 h, and green dots represent fish that survived the 96 h exposure. Regression analyses for the six tissues examined from the 102 fish that died during the 96 h exposure are indicated by the purple line. Regression analyses for the six tissues examined from the 78 fish that survived the 96 h exposure are indicated by the green line.

The relationship between the accumulation rate of Cd in different tissues and the Cd exposure concentration is shown in Figure 3. The Cd accumulation rates in the gills, liver, kidneys, heart, carcass, and the whole body of dead and surviving fish were linearly and positively correlated with the Cd exposure concentrations (*p* ˂ 0.05). Regression analyses for the six tissues examined from the 102 fish that died during the 96 h exposure are *y* = 0.31*x* − 1.8, r^2^ = 0.89, *p* < 0.001 (gill); *y* = 0.14*x* − 0.58, r^2^ = 0.91, *p* < 0.001 (liver); *y* = 0.60*x* − 3.1, r^2^ = 0.91, *p* < 0.001 (kidney); *y* = 0.060*x* − 0.35, r^2^ = 0.92, *p* < 0.001 (heart); *y* = 0.021*x* − 0.12, r^2^ = 0.90, *p* < 0.001 (carcass); *y* = 0.027*x* − 0.15, r^2^ = 0.90, *p* < 0.001 (whole fish). Regression analyses for the six tissues examined from the 78 fish that survived the 96 h exposure are *y* = 0.011*x* + 0.0081, r^2^ = 0.95, *p* < 0.001 (gill); *y* = 0.049*x* − 0.11, r^2^ = 0.69, *p* < 0.05 (liver); *y* = 0.046*x* − 0.035, r^2^ = 0.74, *p* < 0.05 (kidney); *y* = 0.0036*x* − 0.010, r^2^ = 0.74, *p* < 0.05 (heart); *y* = 0.0012*x* − 0.0015, r^2^ = 0.91, *p* < 0.05 (carcass); *y* = 0.0018*x* − 0.0019, r^2^ = 0.95, *p* < 0.05 (whole fish), where *x* is the Cd exposure concentration and *y* is the Cd accumulation rate for the respective tissues.

The slope of the Cd accumulation rate in different tissues and the whole body of dead fish with the Cd exposure concentration was significantly higher than that for the surviving fish (gill *F* = 149.001, *p* < 0.001; liver *F* = 33.025, *p* < 0.001; kidney *F* = 169.770, *p* < 0.001; heart *F* = 71.909, *p* < 0.001; carcass *F* = 143.405, *p* < 0.001; whole fish *F* = 175.555, *p* < 0.001). The effect of Cd exposure concentration on the accumulation rate of Cd in different tissues and the whole body of dead and surviving fish was also very significant (gill *F* = 168.796, *p* < 0.001; liver *F* = 90.655, *p* < 0.001; kidney *F* = 219.104, *p* < 0.05; heart *F* = 82.345, *p* < 0.001; carcass *F* = 199.249, *p* < 0.001; whole fish *F* = 219.440, *p* < 0.001).

### 3.3. Carcass Na^+^ and Ca^2+^ Status

**Figure 4:**
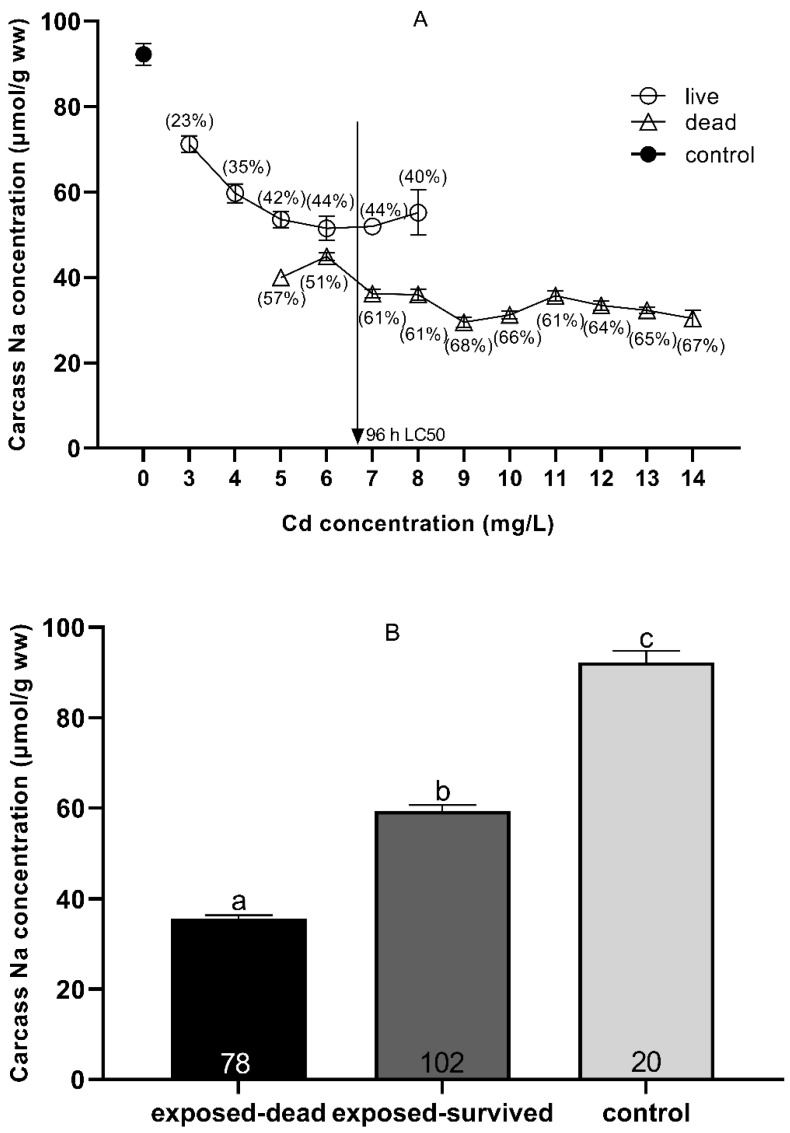
Carcass Na^+^ (means ± SEM) in *Silurus meridionalis* sampled at death due to Cd exposure (**A**). The squares, circles, and triangles represent Na^+^ levels in the control, live, and dead fish, respectively. The percentages represent carcass Na^+^ losses in live and dead fish relative to the control fish. The vertical arrow shows the respective 96 h LC_50_. In some cases, SEMs cannot be seen as they are smaller than the symbol used to indicate the mean. Carcass Na^+^ concentration in the control, exposed-surviving, and exposed-dead fish across all treatments (**B**). Data are given as the mean ± SEM. Sample size, n, was 20, 102, and 78 for the control, dead, and live fish, respectively. Means with different superscript letters a, b, c differ significantly (*p* < 0.05).

The concentrations of Na^+^ ions in the control (0 mg Cd/L) and the carcasses of dead and surviving fish after acute Cd exposure are shown in Figure 4. After Cd exposure, the concentration of Na^+^ ions in the surviving fish decreased by 23–44%, while the concentration in the dead fish decreased by 51–68%, as compared with the concentration of Na^+^ ions in the carcasses of the controls (0 mg Cd/L). When the Cd exposure concentration was 96 h LC_50_, the Na^+^ ion concentration suddenly decreased to 61% (Figure 4A). Although the concentration of Na^+^ ions (59.34 ± 1.29 μmol/g wet weight) of all the surviving fish after Cd exposure was significantly higher than that of all the dead fish (34.54 ± 0.56 μmol/g wet weight) (*p* < 0.001, *t*-test), the concentration of Na^+^ ions was significantly lower than that of the control (0 mg Cd/L) (92.24 ± 2.51 μmol/g wet weight) (*p* < 0.001, *t*-test) (Figure 4B).

**Figure 5:**
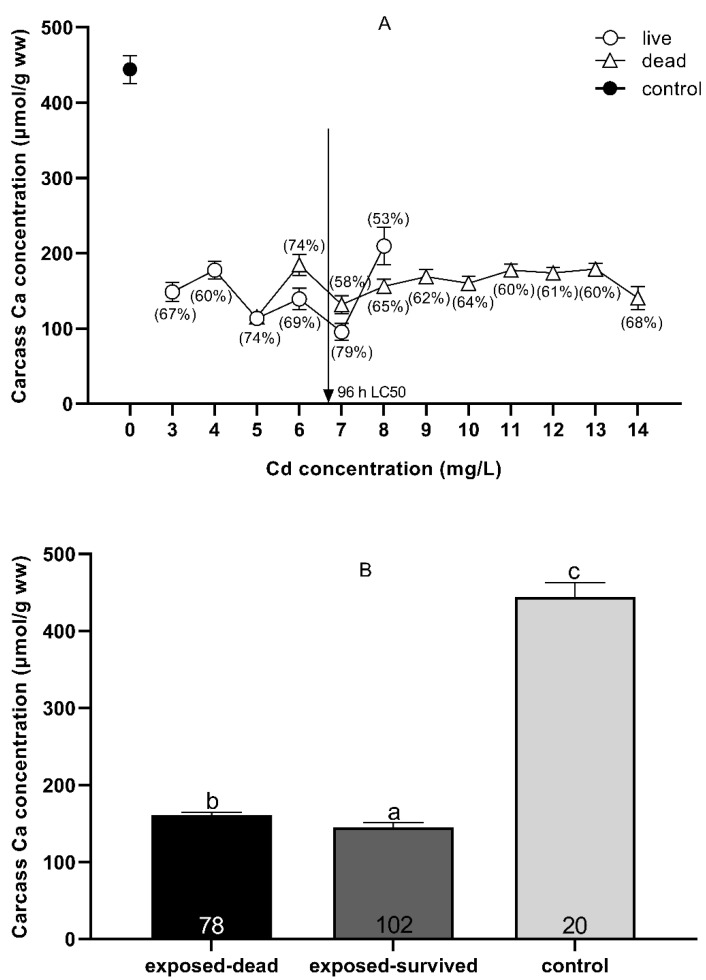
Carcass Ca^2+^ (means ± SEM) in *Silurus meridionalis* sampled at death due to Cd exposure (**A**). The squares, circles, and triangles represent Ca^2+^ levels in the control, live, and dead fish, respectively. The percentages represent carcass Ca^2+^ loss in live and dead fish relative to the control fish. The vertical arrow shows the respective 96 h LC_50_. In some cases where SEMs cannot be seen, they are smaller than the symbol used to indicate the mean. The carcass Ca^2+^ concentration in the control, exposed-surviving, and exposed-dead fish across all treatments (**B**). Data are given as the mean ± SEM. Sample size, n, was 20, 102, and 78 for the control, dead, and live fish, respectively. Means with different superscript letters a, b, c differ significantly (*p* < 0.05).

The concentrations of Ca^2+^ ions in the control (0 mg Cd/L) and the carcasses of dead and surviving fish after acute Cd exposure are shown in Figure 5. There was no clear boundary between the concentration of Ca^2+^ ions in the dead and surviving fish after Cd exposure (Figure 5A). Comparison of the overall mean Ca^2+^ concentration of the carcass showed that the Ca^2+^ concentration (160.67 ± 3.89 μmol/g wet weight) of all the dead fish after Cd exposure was significantly higher than that of all the surviving fish (145.01 ± 5.99 μmol/g wet weight) (*p* = 0.030, *t*-test), but significantly lower than the carcass Ca^2+^ concentration of all controls (0 mg Cd/L) (444.08 ± 18.43 μmol/g wet weight) (*p* = 0.000, *t*-test) (Figure 5B).

## 4. Discussion

The Cd content and accumulation rate of different organs and the whole fish of southern catfish increased with the increase of the Cd exposure concentration, but there were obvious differences between dead and surviving individuals. The results showed that under the same Cd exposure concentration, the Cd content in the gills, kidneys, heart, carcass, and whole fish of southern catfish that died during the exposure was significantly higher than that of the surviving individuals, which was consistent with the higher Cd accumulation rate in the dead individuals. Toxic effects occur when the rate of accumulation of heavy metals exceeds the combined rate of detoxification and excretion of heavy metals [57]. The results of our study suggest that the accumulation rate of Cd is related to lethal toxicity.

The liver Cd content of dead southern catfish firstly increased with the increase of the Cd exposure concentration and then tended to be stable, staying within a small range of change (the mean value was 19.79 μg/g wet weight, about 99 μg/g dry weight, and liver water content was 80%). This suggests that the accumulation of Cd in the liver reached saturation. Studies have shown that the liver tissue of carp (*Cyprinus carpio*) has a limited accumulation capacity for Cd and reaches saturation when the accumulation concentration of Cd reaches 90 μg/g dry weight [58]. This value is almost the same as the Cd accumulation saturation concentration in the livers of southern catfish in the current study. Generally, the liver is thought to be the first site of Cd storage; it not only acts as a storage organ but also as the primary site for the detoxification mechanisms of Cd [59]. The accumulation concentration of Cd in the liver reached saturation, reflecting the limited detoxification ability of the fish liver to respond to Cd. Cd may be bound to metallothioneins in the liver, then transported to the kidneys, which are the main storage site for Cd [60]. The amount of Cd in the kidneys also reached saturation [58]. Although the accumulation concentration of Cd was highest in the kidneys of the dead fish in the current study, it did not reach saturation level. Thus, it can be supposed that after the Cd accumulation concentration in the livers of southern catfish reached saturation, there was a relative increase in Cd transport from the liver to the kidneys, and an increase in direct Cd intake into the kidneys from the blood. Moreover, the high Cd exposure concentration in our study might have caused the accumulation of Cd in the liver to reach saturation.

There was no overlap of whole-fish Cd accumulation concentrations for the southern catfish in the dead and surviving individuals; instead, there was a clear threshold (Figure 2), suggesting that there is a lethal threshold concentration for Cd accumulation in whole fish (1.38 μg/g wet weight). The lethal threshold for Cd exposure in fish was only found for the Cd concentration in the gills [61] and in whole fish [62]. Previous studies on Cd toxicity of 31 different species of fish have shown that the whole-fish Cd concentration, which causes sublethal and lethal toxicity responses, occurs between 0.1 and 4 μg/g wet weight [34]. In our study, the lethal threshold of Cd concentration in southern catfish was determined as 1.38 μg/g wet weight, which is within the range of Cd concentrations indicated in the above-mentioned study. The threshold concentration obtained in this study for lethal toxicity due to acute Cd exposure of southern catfish may have important ecological and toxicological significance for environmental monitoring of the Cd concentration in fish [63]. No lethal threshold of Cd concentration was found in the tissues of the fish in the current study. After exposure to Cd, metallothionein (MT) expression is induced in the gills, liver, and kidneys, which binds with Cd in detoxification [64]. A toxicity effect occurs when the concentration of free heavy metal Cd ions exceeds the binding capacity of metallothionein [65]. Since only modest changes in whole fish concentrations of MT occur with exposure to metals, whole-fish Cd concentrations are preferred for assessing potentially toxic concentrations of Cd in fish [34]. The results of this study also support the view that the whole-fish Cd concentration is a more suitable surrogate for the effective concentration of toxicity [27].

The bioconcentration factor (BCF) was used to estimate the capacity of the fish to accumulate an element in its tissues [56,66]. The higher BCF values observed for the gill, liver, and kidney tissues reflected their high capacities for the accumulation of Cd present in a contaminated environment. Unlike other studies, our observations indicate that the BCF for Cd do not decrease with increasing exposure [66,67], except for BCF in the liver of dead fish. All BCF calculated in the study of *Channa punctatzcs* acute Cd poisoning [68] indicate that BCF in the gill, liver, and kidney tissues do not decrease with increasing exposure. A possible explanation for this difference is that they considered experimental exposure data to be acceptable only when the exposure duration was at least 28 d for fish [66], whereas in our study, the exposure concentrations were high and the exposure duration was short. Although BCF are often used to assess hazard risks, this method has received scrutiny for its application as a metric for metals [15,66,69,70]. To be a representative property of a contaminant, the BCF should be constant across different exposure conditions and species [15]. BCF in carcass and whole fish in the current study tended to be stable as the slopes neared zero, which indicate that these BCF can be useful in acute exposure conditions. All BCF calculated in this study are higher than channel catfish (*Ictalurus punctatus*) chronic exposure to environmental Cd [69].

The results show that acute Cd exposure led to the loss of Ca and Na ions in the southern catfish, and that the rate of Na ion loss in the carcass of dead fish was significantly higher than that in the surviving fish (Figure 4), which indicates that the lethal toxicity of acute Cd exposure is related to the loss of Na ions. A large number of studies have shown that exposure to Cd inhibits carbonic anhydrase and Na^+^/K^+^-ATPase activity in the gills and kidneys [39,44,46] and induces endocrine stress responses [14,41], leading to a loss of Na ions in fish that kills the fish by causing the cardiovascular system collapse [71,72]. The consistency of the whole-body Na^+^ content of dead fish across all exposure concentrations (Figure 4) suggests that there is a critical Na^+^ level, circa 34.54 μmol/g wet weight, below which survival is no longer possible. Our findings provide new evidence supporting the research of Veltman et al. (2014), who concluded that when the Na ion concentration in the body of freshwater aquatic organisms is below a certain threshold, it leads to death [42]. Our results are also consistent with the conclusion that the significance of the exposure of zebrafish (*Danio rerio*) to Cd is related to a threshold of Na ion concentration [73].

The acute toxicity mechanism resulting from exposure of fish to Cd is the disruption of Ca ion homeostasis [36,45]. Water Cd^2+^ and Ca^2+^ compete for apical Ca^2+^ channels in the gill tissue [74], Cd^2+^ inhibits basolateral Ca^2+^-ATPase activity in the gills [75], and Cd^2+^ inhibits Ca^2+^-ATPase activity in the muscles [76]. Although exposure to Cd resulted in a decrease in the concentration of Ca ions in the southern catfish, the overall mean value of Ca ion concentration in the carcasses of dead fish was higher than the Ca ion concentration in the carcasses of surviving fish (Figure 5), indicating that the decreased Ca ion concentration in the carcass was not correlated with the death of the southern catfish resulting from acute Cd exposure. In addition, we should also note that at very high waterborne Cd concentrations at a lethal concentration, high concentrations of metals may accumulate on the respiratory surface and prevent the respiratory gas exchange, thus leading to suffocation [28].

## 5. Conclusions

In conclusion, the Cd content and accumulation rate in different organs and the whole fish of the southern catfish were positively correlated with Cd exposure concentration, and there were obvious differences between dead and surviving individuals. The quantitative relationships between Cd accumulation concentration in different tissues of fish and Cd exposure concentrations was provided in the current study under acute exposure conditions. These results indicated that both the Cd content of tissues and Cd accumulation rates were correlated with mortality. The Cd content of the liver of dead fish reached saturation (19.79 μg/g wet weight), and the Cd content of the kidneys increased with the increase in Cd exposure concentration, indicating that the detoxification ability of the liver of southern catfish was limited. There is a lethal threshold concentration (1.38 μg/g wet weight) for the accumulation of Cd in whole fish, which can be used as an indicator of environmental toxicology. Unlike other studies, our observations indicate that the BCF for Cd do not decrease with increasing exposure. The lethal toxicity to southern catfish resulting from acute Cd exposure is related to a decrease in the Na ion concentration, while decreased Ca ion concentrations in the carcass are not correlated with acute exposure to Cd that lead to the death of the southern catfish. The present work provides preliminary insights into the possible impacts of sudden high levels of Cd contaminants in freshwater ecosystem. The results suggest that whole-fish Cd content and carcass Na^+^ content are useful indicators of fish acutely exposed to Cd.

Fish can uptake waterborne and foodborne Cd. Fish have high affinity to Cd. Metals are not degradable but rather accumulate in the environment, and due to the biomagnification effect, they move up the food chain, progressively increasing their concentrations in the tissues of fish [7]. This can be a public health concern. In the future, it will be necessary to investigate Cd accumulation and effects when the southern catfish are exposed through environmentally relevant Cd and food.

## Data Availability

Not applicable.
